# Neuropeptides in the Extracellular Space of the Mouse Cortex Measured In Vivo by Nanodialysis Probe Coupled with LC‐MS

**DOI:** 10.1002/anie.202509490

**Published:** 2025-08-11

**Authors:** Keyin Li, Weihua Shi, Yanqi Tan, Yu Ding, Alex G. Armstrong, Yurii Vlasov, Jonathan V. Sweedler

**Affiliations:** ^1^ Neuroscience Program University of Illinois Urbana‐Champaign Urbana IL 61801 USA; ^2^ Department of Electrical and Computer Engineering University of Illinois Urbana‐Champaign Urbana IL 61801 USA; ^3^ Department of Physics University of Illinois Urbana‐Champaign Urbana IL 61801 USA; ^4^ Department of Chemistry and Beckman Institute for Advanced Science and Technology University of Illinois Urbana‐Champaign Urbana IL 61801 USA

**Keywords:** In vivo sampling, Mass spectrometry, Microdialysis, Neurochemistry, Neuropeptides

## Abstract

Neuropeptides are key neuromodulators in the central nervous system that shape sensory processing, yet their extracellular dynamics in the somatosensory cortex (S1) remain poorly understood. This study applies an innovative membrane‐free silicon nanodialysis (ND) probe coupled with liquid chromatography‐mass spectrometry (LC‐MS) to analyze the extracellular neuropeptidome in the mouse S1 with spatial resolution down to 100 µm. Localized in vivo sampling identified extracellular peptides from secretogranin‐1, ProSAAS, pro‐opiomelanocortin (POMC), and others. Minimal tissue damage, enabled by probe dimensions of 75 × 15 µm^2^, resulted in an absence of structural peptides in the dialysate indicating low intracellular contamination. Many detected secretory peptides correlated with strong local mRNA expression; however, the detection of POMC‐derived peptides, despite negligible local expression, suggests long‐distance peptide transport or extracellular processing. To expand peptide identification, a discovery‐to‐targeted peptidomic approach was developed, revealing 46 peptides from 24 proteins in dialysate samples, including 10 proteins with low local expression. Complementary S1 tissue analysis confirmed POMC peptides and showed that 17% of 304 prohormone‐derived peptides had low local expression. These results uncover a complex extracellular peptide landscape shaped by both local and long‐distance signaling. By overcoming the limitations of traditional microdialysis, this approach advances the understanding of neuropeptide signaling in cortical function.

## Graphical abstract

This study reveals a complex peptidomic landscape in the mouse somatosensory cortex, identifying locally released and potentially transported neuropeptides using a membrane‐free nanodialysis sampling probe coupled with nanoLC‐timsTOF mass spectrometry.

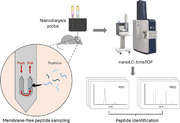

## Introduction

Neuropeptides are a diverse set of cell‐cell signaling molecules in the central nervous system (CNS) that can act as neurotransmitters,^[^ ^1^ ^]^ but mostly as neuromodulators and as hormones.^[^ ^2^ ^]^ These peptides regulate a wide range of physiological processes, including behavior, energy balance, and pain perception. For example, substance P mediates pain transmission and is released by sensory neurons in response to noxious stimuli^[^ ^3^ ^]^; neuropeptide Y (NPY) and agouti‐related peptide (AgRP) are involved in appetite and feeding behaviors.^[^ ^4^ ^]^ The somatosensory cortex is a critical center for sensory integration. Although evidence suggests several neuropeptides modulate its circuits,^[^ ^5^, ^6^, ^7^ ^]^ its extracellular neuropeptide landscape remains poorly defined. Investigating the extracellular neuropeptidome with high spatial resolution is essential for uncovering the roles these molecules play in cortical function and dysfunction.

Despite their importance, studying extracellular peptides presents several challenges. Unlike classical neurotransmitters that primarily act through fast synaptic transmission, neuropeptides often exert their effects through volume transmission, diffusing away from their release location within the extracellular space to influence a larger number of target cells. Depending on the chemical structure of the peptides, their half‐lives after release vary. The peptides released by neurons may act within a few microns of the release site, while others have longer extracellular half‐lives that maintain activity during long‐distance diffusion.^[^ ^2^, ^8^ ^]^ This diffusion process, along with the dynamic extracellular modifications that neuropeptides undergo, plays a key role in their biological activity. Adding complexity and subtlety to their actions, receptor binding, and peptide half‐life depend on their exact molecular form. Besides the primary sequence of amino acids, they incorporate numerous possible post‐translational modifications (PTMs) that occur within the intracellular secretory pathway, as well as enzymatic cleavages and other modifications within the extracellular fluid after secretion. These changes can generate bioactive peptides from the same precursor that have distinct functions. For example, prohormone cleavages usually occur at mono‐ and dibasic amino acid residues by endoproteases.^[^ ^9^ ^]^ In addition to these sites, several peptides have been found to arise from cleavage at nonbasic processing sites,^[^ ^10^, ^11^ ^]^ and may be formed by extracellular peptidases. The dynamics of enzymatic processing in the extracellular fluid of distinct brain areas are still being explored.^[^ ^12^ ^]^ Lastly, prohormones expressed in a soma can be transported within dense core vesicles long distances, so the final bioactive peptides can be released in locations far removed from their initial locations of synthesis. Combined, these characteristics highlight the need for reliable in vivo methods to sample peptides from the extracellular space at precise locations to precisely define the neuropeptidome at specific locations and under specific conditions.

Existing methodologies for in vivo neuropeptide detection have several limitations that restrict their effectiveness. While direct tissue analysis can provide details on the forms of peptides at a specific location, it fails to distinguish between intra‐ and extracellular pools.^[^ ^12^ ^]^ Microdialysis remains a widely used approach for monitoring peptides in vivo due to its ability to sample extracellular fluid directly. However, microdialysis faces challenges when applied to larger molecules like peptides (as compared to the classical neurotransmitters) that include low recovery rates, non‐specific adsorption to membranes, and low concentration of the peptides in the brain extracellular fluid (making their measurement problematic).^[^ ^13^ ^]^ As neuropeptides are 3–100 amino acids long, larger than classic neurotransmitters,^[^ ^14^ ^]^ they also have smaller diffusion rates. Most of the relative recoveries with microdialysis probes are reported to be lower than 20%,^[^ ^15^, ^16^, ^17^ ^]^ although some recently developed novel sampling approaches achieved >80% recoveries in vitro.^[^ ^18^, ^19^ ^]^ Even for these later probes, the in vivo recovery may be lower as the diffusion of peptides is primarily affected by tortuosity in the surrounding brain tissue.^[^ ^20^ ^]^ Therefore, it would be beneficial to have a probe with a smaller area of analyte depletion (penetration depth) with higher peptide recovery. In addition, the relatively large sizes of the conventional microdialysis probe (0.2–0.5 mm in diameter) cause damage in the surrounding tissue and could result in an inflammatory response with the formation of glial scarves, which provide diffusion barriers for peptides.^[^ ^15^ ^]^ Another approach is the push‐pull capillary; push‐pull systems bypass some of these issues. For example, the recovery of push‐pull perfusion is higher because of the unrestricted analyte exchange between perfusate and extracellular fluid. However, the convection flow in this configuration may lead to shear distress of surrounding tissue, as well as potential clogging of the pull channel by tissue debris.

In addition, samples collected by microdialysis or push‐pull perfusion contain low concentrations of peptides present in small sample volumes. Neuropeptides are present in extracellular fluids at low concentrations in nM to pM range,^[^ ^21^ ^]^ requiring the detection of less than 1 femtomole of peptides. Mass spectrometry (MS) detection provides excellent limits of detection and the ability to detect a wide range of analytes when compared to immunoassay‐based detection methods.^[^ ^22^ ^]^ In the last decade, many studies have used liquid chromatography MS (LC‐MS) to measure targeted neuropeptides in brain dialysate samples, including enkephalins,^[^ ^23^, ^24^, ^25^ ^]^ β‐endorphin,^[^ ^26^ ^]^ oxytocin,^[^ ^27^ ^]^ angiotensins,^[^ ^28^ ^]^ neuropeptide Y,^[^ ^29^ ^]^ and substance P.^[^ ^30^ ^]^ Several previous studies also reported using peptidomic approaches to discover novel endogenous species.^[^ ^31^, ^32^, ^33^ ^]^ Our major goal is to downscale these systems to allow precise sampling from specific brain regions, discovering previously uncharacterized extracellular neuropeptides and gaining deeper insights into their spatial distribution, processing, and functional roles in cortical signaling. By coupling a nanodialysis (ND) probe with LC‐MS, we identify a range of neuropeptides derived from secretory proteins and prohormones, shedding light on their potential sources and extracellular transport mechanisms. This work demonstrates the utility of high‐resolution sampling for peptidergic research and provides new insights into neuropeptide dynamics in the cortex.

## Results and Discussion

### The Design and Fabrication of the Membrane‐Free Silicon Platform

**Figure 1:**
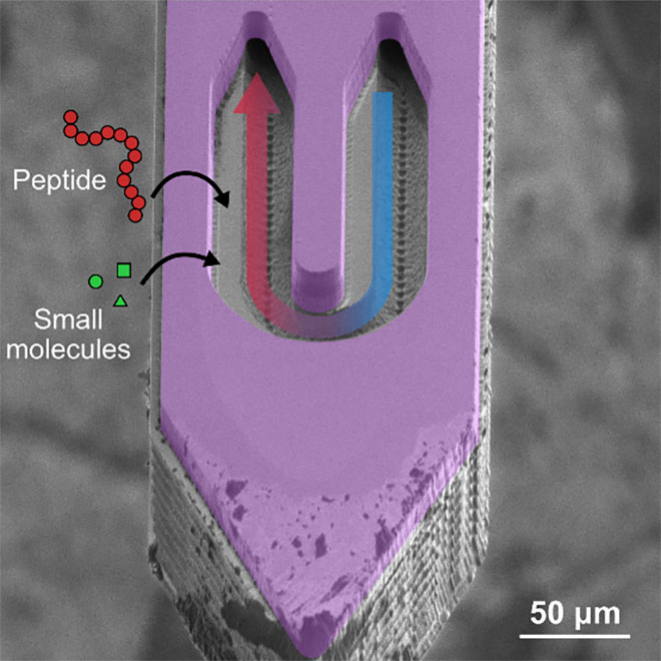
Scanning electron microscope (SEM) image of the nanodialysis probe tip, illustrating the open sampling area where balanced in‐plane flow facilitates diffusion‐driven analyte collection from brain extracellular fluid.

The 15 µm‐thin ND probe is made from silicon, with an embedded microfluidic channel of 7‐µm radius. Details of the microfabrication are provided in our previous work.^[^ ^34^ ^]^ The ND probe has a 20 x 100 µm^2^ U‐shaped open sampling area located at the tip of the needle (Figure 1). Although our probe has a much smaller sampling area, we employ an ultraslow flow rate to ensure sufficient collection of peptides taking into account typical peptide diffusion rates. The operation of the ND probe is similar to a conventional push‐pull design, with inlet and outlet channels connected to two pressure pumps. However, the U‐shaped open channel facilitates a balanced in‐plane flow under a flow rate of 10 nL min^−1^, enabling purely diffusion‐driven analyte collection.^[^ ^35^ ^]^ This design largely reduces tissue damage associated with probe insertion and sampling process, limiting tissue dislocation to the probe's dimensions with no flow‐induced damage. A comprehensive analysis of tissue damage associated with the ND probe is described in our prior study.^[^ ^36^ ^]^ The minimal disruption ensures that extracellular components are sampled with minimal contamination from intracellular peptides. Additionally, the depletion depth of the probe is also reduced, which minimizes the impact of tissue tortuosity on peptide recovery, leading to improved sampling efficiency and a more accurate representation of extracellular peptide concentrations.

Furthermore, the membrane‐free design provides significant benefits over membrane‐based sampling probes, especially for the collection of larger molecules. Membrane‐based probes hinder the diffusion of larger analytes due to their size‐exclusion effects and therefore reduce the efficiency of peptide collection. While molecules smaller than the molecular weight cut‐off can diffuse through the membrane into the sampling area, membranes still introduce diffusion resistance that slows the transfer, reducing the recovery rate. Moreover, the use of membranes can contribute to non‐specific adsorption or fouling, a common problem that may compromise sampling efficiency or impact analyte integrity. By eliminating the membrane, our ND probe allows unimpeded diffusion of peptides into the sampling area. This design reduces diffusion resistance and minimizes the risk of analyte loss due to adsorption or fouling of the membrane, enhancing the platform's ability to provide a more comprehensive and representative peptide profile of the extracellular environment.

In vitro characterization demonstrated that the entire sampling workflow has a relative recovery of approximately 20% for three neuropeptide standards: angiotensin II, Leu‐enkephalin, and Met‐enkephalin (Figure S1A). Most peptide loss occurs due to adsorption on the storage capillaries and during sample transfer, as indicated by control experiments showing similar recovery rates when extracting peptide standards from storage capillaries (Figure S1B). This suggests that sampling loss on the ND probe itself is minimal. The observed ∼20% recovery is on par with most of previously reported microdialysis‐based peptide sampling methods, ranging from 1% to 30%.^[^ ^17^, ^26^, ^37^, ^38^ ^]^

### Detecting Neuropeptides in Mouse Somatosensory Cortex

**Figure 2:**
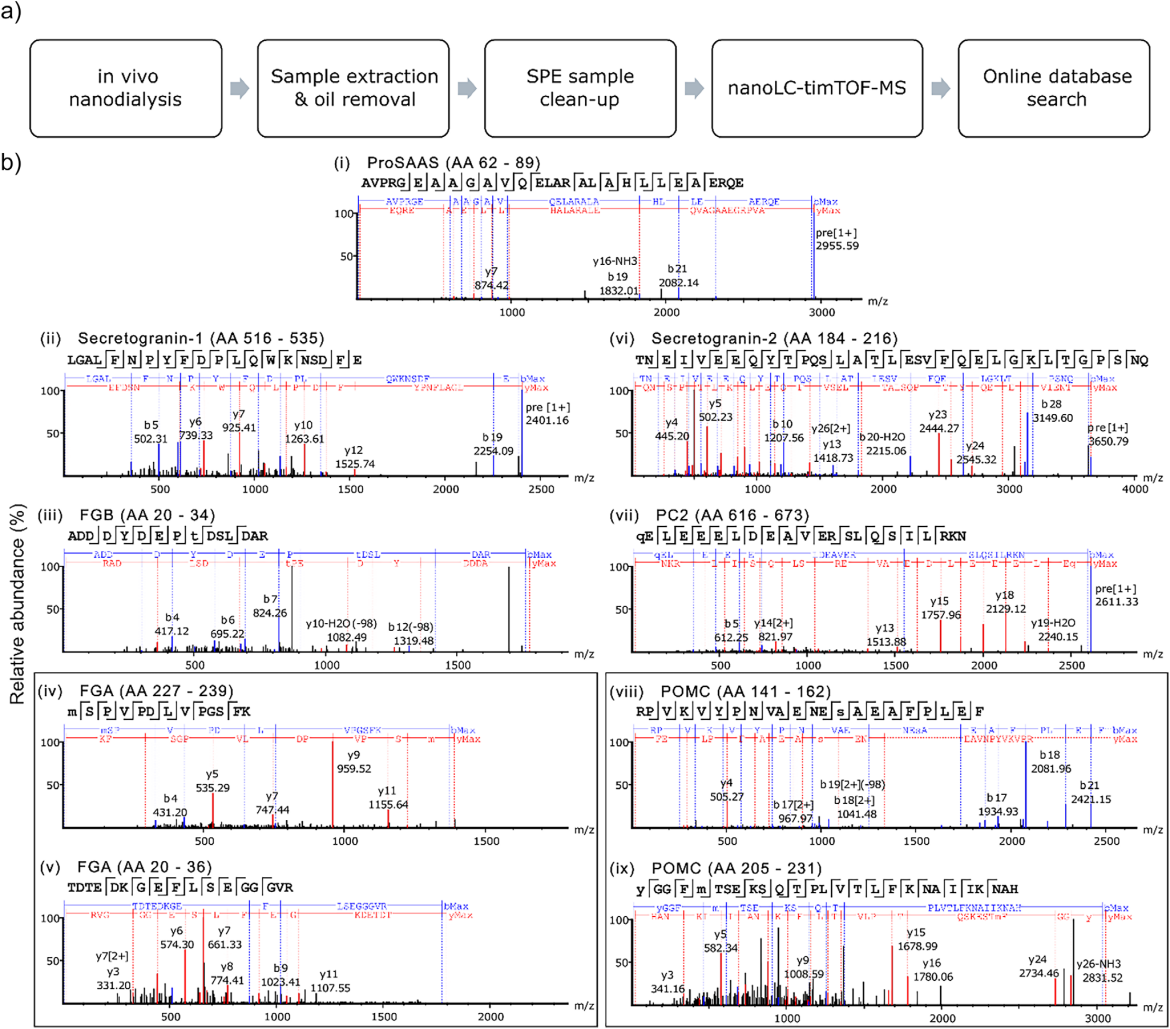
Peptide identification from in vivo dialysate sample using DDA‐PASEF MS analysis. a) Overview of the sample preparation and peptide identification workflow. b) Representative MS/MS spectra of peptides identified from various proteins with corresponding amino acid (AA) positions: i) ProSAAS, ii) secretogranin‐1, iii) fibrinogen beta, iv, v) fibrinogen alpha, vi) secretogranin‐2, vii) proprotein convertase 2 (PC2), viii, ix) pro‐opiomelanocortin (POMC). Fragment ions identified in the MS/MS spectra are labeled as blue (b ions) and red (y ions).

Understanding the composition of extracellular peptides in the brain is crucial for deciphering their functions in signaling. To investigate these peptides in the mouse cortex, we performed in vivo sampling from the somatosensory cortex of an anesthetized mouse. We implanted the ND probe into layer 4 of the primary somatosensory area (S1). A 1 µL dialysate sample was collected and analyzed by LC‐MS with a data‐dependent acquisition parallel accumulation‐serial fragmentation (DDA‐PASEF) method. A schematic of the experimental workflow after in vivo sampling is shown in Figure 2a.

A total of 22 peptides derived from 7 different protein precursors were identified in this dialysate sample. These included 2 peptides from secretogranin‐1 (SgI), 1 peptide from secretogranin‐2 (SgII), 1 peptide from ProSAAS, 2 peptides from fibrinogen alpha, 1 peptide from fibrinogen beta, 14 peptides from pro‐opiomelanocortin (POMC), and 1 peptide from proprotein convertase 2 (PC2) (Table 1). Several POMC‐derived peptides were detected with PTMs, suggesting their potential biological relevance. Representative MS/MS spectra for these peptides are shown in Figure 2b. All of the identified peptides are derived from secretory proteins. For example, SgI, SgII, and ProSAAS are widely expressed across the CNS, including neuroendocrine and neuronal cells.^[^ ^39^, ^40^ ^]^ They are known precursors of a wide range of bioactive peptides involved in diverse physiological and pathophysiological conditions, including CgB_1‐41_ (from SgI),^[^ ^41^ ^]^ secretoneurin (from SgII),^[^ ^42^ ^]^ and big LEN (from ProSAAS).^[^ ^43^ ^]^ Although the specific peptides detected from these proteins in this study have not been reported as bioactive, their presence appears to reflect intermediates or incompletely processed peptides released into the extracellular space.

**Table 1:** List of identified peptides from the mouse brain dialysate.

Peptide	Protein name	−10LgP	Area	Mass	ppm	m/z	z	PTM with Ascore
MSPVPDLVPGSFK	Fibrinogen alpha	30.9	23 290	1388.701	−8	695.3552	2	M1:Oxidation: 1000
TDTEDKGEFLSEGGGVR	Fibrinogen alpha	35.91	25 278	1795.822	−7	599.6132	3	
ADDDYDEPTDSLDAR	Fibrinogen beta	27.01	2572.8	1776.636	−8.1	889.3221	2	T9:Phosphorylation: 0.00
RPVKVYPNVAENESAEAFPLE	Pro‐opiomelanocortin	26.8	7307.5	2438.152	4.1	813.7315	3	S14:Phosphorylation: 19.61
RPVKVYPNVAENESAEAFPLEF	Pro‐opiomelanocortin	35.92	768.02	2504.27	−6.5	835.7621	3	F22:Amidation: 1000
RPVKVYPNVAENESAEAFPLEF	Pro‐opiomelanocortin	53.37	584 320	2585.22	3.2	862.7539	3	S14:Phosphorylation: 47.82
RPVKVYPNVAENESAEAFPLEF	Pro‐opiomelanocortin	32.64	1214.7	2584.236	−11.9	862.413	3	S14:Phosphorylation: 32.94; F22:Amidation: 1000
RPVKVYPNVAENESAEAFPLEF	Pro‐opiomelanocortin	35.95	584 320	2585.22	−4.5	862.7473	3	Y6:Phosphorylation: 8.77
RPVKVYPNVAENESAEAFPLEF	Pro‐opiomelanocortin	46.41	99 649	2505.254	−0.4	836.0952	3	
YGGFMTSEKSQTPLVTL	Pro‐opiomelanocortin	50.24	29 827	1899.929	−6	950.9702	2	Y1:Acetylation: 1000
YGGFMTSEKSQTPLVTL	Pro‐opiomelanocortin	41.11	20 427	1915.924	−3.2	958.9703	2	Y1:Acetylation: 1000; M5:Oxidation: 1000
YGGFMTSEKSQTPLVTLF	Pro‐opiomelanocortin	44.76	102 960	2046.997	−7.9	1024.502	2	Y1:Acetylation: 1000
YGGFMTSEKSQTPLVTLF	Pro‐opiomelanocortin	40.6	48 759	2062.992	−1.4	1032.507	2	Y1:Acetylation: 1000; M5:Oxidation: 1000
YGGFMTSEKSQTPLVTLFKNAIIKNA	Pro‐opiomelanocortin	55.9	129 280	2899.515	−0.6	967.5161	3	Y1:Acetylation: 1000
YGGFMTSEKSQTPLVTLFKNAIIKNA	Pro‐opiomelanocortin	49.95	74 785	2915.51	−4.1	972.8443	3	Y1:Acetylation:1000; M5:Oxidation: 1000
YGGFMTSEKSQTPLVTLFKNAIIKNAH	Pro‐opiomelanocortin	49.99	30 788	3036.574	10.8	760.1624	4	Y1:Acetylation: 1000
YGGFMTSEKSQTPLVTLFKNAIIKNAH	Pro‐opiomelanocortin	29.66	7616.8	3052.569	0.5	764.1533	4	Y1:Acetylation: 1000; M5:Oxidation: 1000
QELEEELDEAVERSLQSILRKN	Proprotein convertase 2	36.86	3151.6	2610.314	−1.4	871.1145	3	Q1:Pyro‐glu from Q: 1000
AVPRGEAAGAVQELARALAHLLEAERQE	ProSAAS	50.21	10 693	2954.569	1.1	739.6535	4	
GALFNPYFDPLQWKNSDFE	Secretogranin‐1	30.15	50 093	2287.059	2.5	1144.544	2	
LGALFNPYFDPLQWKNSDFE	Secretogranin‐1	61.88	6131.5	2400.143	2.7	1201.087	2	
TNEIVEEQYTPQSLATLESVFQELGKLTGPSNQ	Secretogranin‐2	68.03	40 527	3649.8	10.8	1217.626	3	

Fibrinogen is a key plasma protein involved in coagulation,^[^ ^44^ ^]^ and is traditionally thought to be excluded from the brain parenchyma by the blood‐brain barrier (BBB).^[^ ^45^ ^]^ However, recent studies suggest more complex dynamics. While its presence in the CNS has often been linked to vascular injury, emerging evidence indicates local expression: in situ hybridization (ISH) from the Allen Mouse Brain Atlas shows substantial mRNA expression of fibrinogen chains in the brain, and Golanov et al. reported constitutive expression of fibrinogen proteins in astrocytes and neurons.^[^ ^46^ ^]^ Furthermore, Maki et al. found that fibrinogen α‐chain‐derived peptides are upregulated in the hippocampus following morphine exposure and cognitive tasks, supporting regulated expression under physiological conditions.^[^ ^47^ ^]^ In our study, we detected three fibrinogen derived peptides, two from alpha chain and one from beta chain. While this may reflect minor BBB disruption during probe implantation, its repeated detection under non‐injury conditions in other studies suggests a potential brain‐intrinsic source. Importantly, structural or cytosolic proteins such as actin and tubulin were not detected from this sample. This lack of significant structural protein detection suggests a high specificity of the sampling method for secreted and extracellular proteins while minimizing contamination from intracellular debris or structural components. These results highlight the robustness of the membrane‐free sampling approach for extracellular neurochemical analysis in vivo.

**Figure 3:**
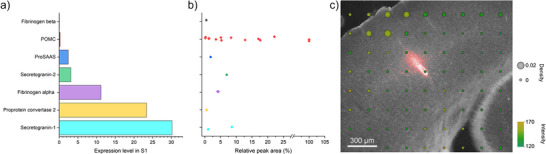
Comparison of peptide profile and protein expression in the sampled area. a) The expression level of proteins corresponds to the identified peptides in the S1 region. b) The relative MS1 peak area of the identified peptides grouped by their protein precursors. c) The histology of the brain cortex and hippocampus overlays with the sampling site (red) and the POMC expression level (dots). The size of the dots indicates the expression density, and the color indicates the expression intensity.

To better understand whether the detected peptides result from local protein secretion or alternative mechanisms such as volume transmission, we queried the local expression levels of their genes in S1 using a web‐based visualization tool, Allen Brain Atlas‐Driven Visualizations (ABADV)^[^ ^48^ ^]^ (Figure 3a). This tool allows us to access the ISH expression data of genes in specific brain regions defined in the ABA, providing expression energy (E) values. These values are calculated as the sum of the intensity of expressing pixels in S1 divided by the sum of all pixels in S1. Among the genes analyzed, SgI exhibits the highest expression energy at about 30, while *Pomc* and *Fgb* are almost silent with E values of 0.25 and 0.04, respectively. The remaining genes fall within a moderate range. For most peptides, the detection aligns well with the local expression levels, as indicated by expression energy. Interestingly, while *Pomc* expression is low in this region, multiple POMC‐derived peptides were identified with high confidence and relatively high abundance as indicated by their MS1 peak areas (Figure 3b). Among them, several post‐translationally modified forms of the corticotropin‐like intermediate lobe peptide (CLIP) were detected. The peptide RPVKVYPNVAENES(p)AEAFPLEF showed phosphorylation at S14 with an AScore of 47.82, indicating high localization confidence. Another phosphorylated variant lacking one residue from the C‐terminus was also identified with modification at S14 but with lower confidence (AScore = 19.61). This phosphorylation site of CLIP has been previously reported in several studies.^[^ ^49^, ^50^, ^51^, ^52^ ^]^ The same sequence with phosphorylation on Y6 was identified with low confidence (AScore = 8.77), which could be a misassignment of the S14 phosphorylation or possibly a sulfation, given the similar mass shift. While tyrosine sulfation is biologically feasible, no studies report it for CLIP, making this a potential artifact or novel finding. This sequence also co‐eluted with the S14‐phosphorylated form, contributing to a prominent combined peak. Several other POMC peptides also showed substantial MS1 signals. This suggests a notable presence of processed POMC products in the extracellular space despite low transcript levels.

Figure 3c further supports this finding by overlaying the histology of the cortex with the sampling site and *Pomc* expression levels. The red area indicates the sampling site in the S1 cortex visualized by rhodamine B added in the perfusate. The mRNA expression map of *Pomc*, adapted from Allen Brain Mouse Atlas ISH data,^[^ ^53^ ^]^ indicates varying density and intensity of *Pomc* mRNA expression with dots of different sizes and colors, emphasizing the sparse expression of POMC in the sampling region.

In adult mice, POMC is primarily synthesized in the arcuate nucleus (ARC) of the hypothalamus and processed into multiple neuropeptides involved in critical functions such as energy balance and stress response.^[^ ^54^ ^]^ These peptides are typically distributed through projections from POMC neurons to various brain regions. Although direct projections from POMC neurons in the ARC or nucleus tractus solitarius (NTS) to the cortex have not been reported in comprehensive anatomical tracing studies,^[^ ^55^ ^]^ it suggests a broad POMC signaling, raising the possibility of indirect modulation. POMC and its derivatives have also been shown to enter the ventricular system and diffuse through cerebrospinal fluid (CSF) to reach distant targets.^[^ ^54^, ^56^, ^57^ ^]^ However, the presence of multiple POMC cleavage products in our samples, including CLIP variants and truncated β‐endorphin (β‐END) variants, may be difficult to reconcile with diffusion‐based transport alone, which typically leads to degradation and dilution. Although the precise source of these peptides remains unclear, their presence raises the possibility of local release from non‐canonical projections. Moreover, the co‐detection of a sequence derived from PC2, which is one of the classical POMC processing enzymes, may indicate local proteolytic processing that contributes to the observed POMC peptides.

All of the β‐END derivatives detected in this sample have N‐terminal acetylation modification. It has been shown that the N‐acetylated forms of β‐END and derivatives has very weak affinity to the opioid receptor,^[^ ^58^ ^]^ but regulate the antinociceptive activity of different opioids.^[^ ^59^ ^]^ Recent study also found that acetyl β‐END 1–26 and β‐END 1–27 modifies association of the sigma type 1 receptor (σ1R) with σ2R, NR1 C1 subunits of *N*‐methyl‐D‐aspartate receptors (NMDARs) and binding immunoglobulin protein (BiP) with slightly different effects.^[^ ^60^ ^]^ In addition, multiple forms of CLIP, including phosphorylated variants, were detected in this sample. CLIP is derived from the adrenocorticotropic hormone (ACTH) region of POMC and has been implicated in modulating neural excitability^[^ ^61^ ^]^ and stress responses,^[^ ^62^ ^]^ although its precise functions remain poorly defined. The presence of CLIP and β‐END derivatives with specific PTMs in the cortical extracellular space suggests that these peptides may participate in previously unrecognized mechanisms of cortical signaling or neuromodulation.

### Expanding the Peptidome: Identifying Prohormone‐Derived Peptides and Their Potential Origins in S1

**Figure 4:**
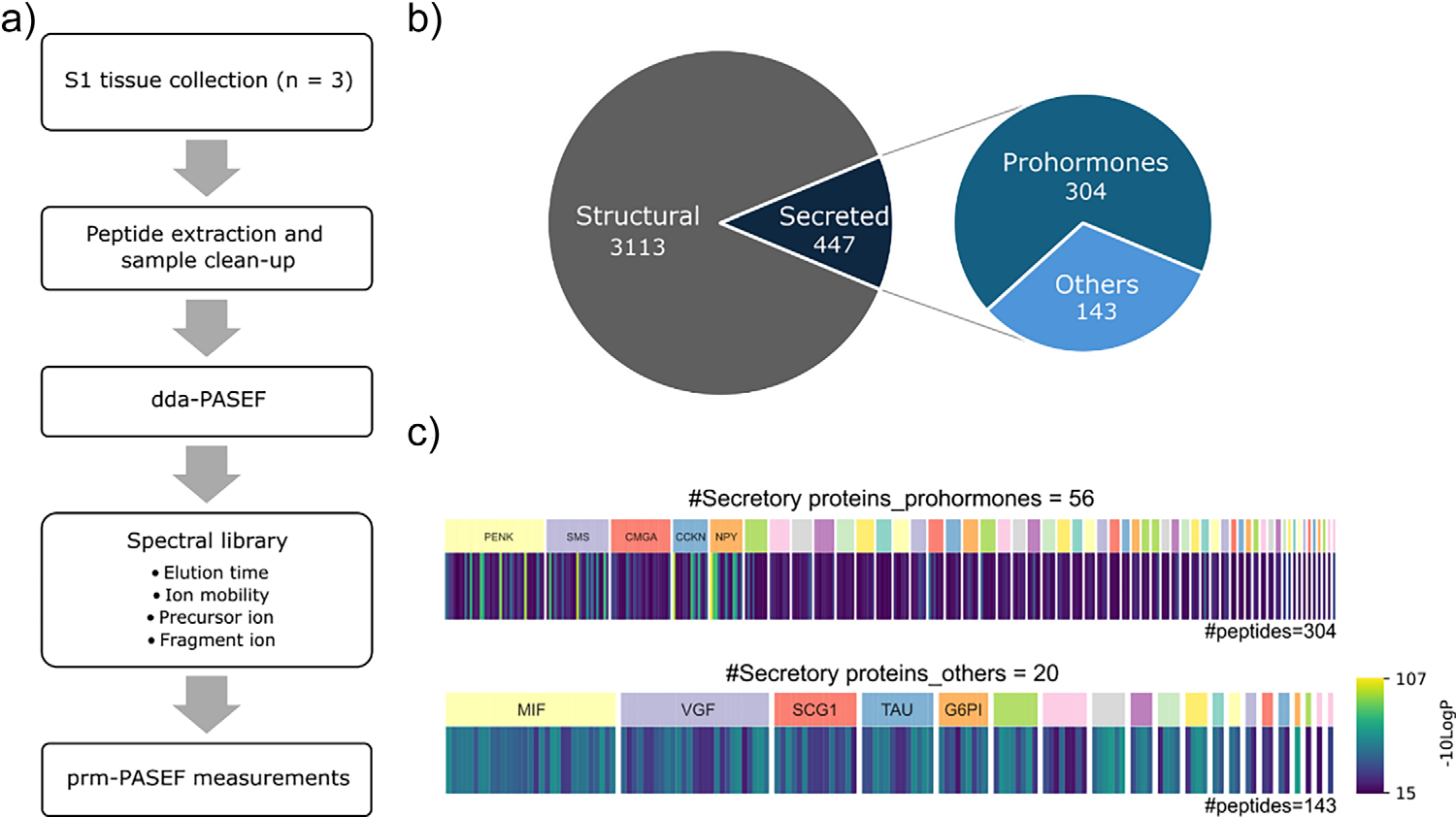
Primary somatosensory (S1) peptidome analysis and spectral library generation. a) Overview of the strategy to generate a PRM‐PASEF method for targeted analysis of extracellular peptides in dialysate samples. b) Distribution of identified peptides from the S1 peptidome. Peptides are categorized into structural (3113 peptides) and secreted (447 peptides) proteins, with the secreted category further divided into prohormones (304 peptides) and others (143 peptides). Peptides from secreted proteins are selected into the spectral library for the following PRM‐PASEF analysis. c) Heatmaps of ‐10LogP values for identified peptides from secretory proteins. Colored blocks above the heatmaps indicate the protein source. The top heatmap represents the prohormone group (304 peptides, 56 proteins), and the heatmap represents other secretory proteins (143 peptides, 20 proteins).

Building on our initial detection of POMC‐derived peptides, we sought to explore whether additional prohormone‐derived peptides—including those potentially conveyed by bulk transfer mechanisms—could be identified in the S1 region. To achieve this, we implemented a targeted peptide analysis designed to enhance sensitivity and selectivity for extracellular neuropeptides. This approach leverages a tissue‐based peptide library to provide a more comprehensive characterization of putative peptides in our target sampling region. The overall workflow for method development is shown in Figure 4a. Tissue samples were collected from the S1 region (∼12 mm^3^) from three mice, pooled, and analyzed by LC‐MS/MS using a DDA method. To maximize neuropeptide discovery, the tissue sample was searched against both the entire mouse protein database^[^ ^63^ ^]^ and a list of prohormones.^[^ ^64^ ^]^ This approach identified 3560 peptides, including 3113 from structural proteins and 447 from secreted proteins, of which 304 were prohormone‐derived (Figure 4b). Figure 4c presents heatmaps of the ‐10LgP scores for identified secretory proteins, categorized as prohormones and other secreted proteins, with all peptides having ‐10LgP scores above 15.

**Figure 5:**
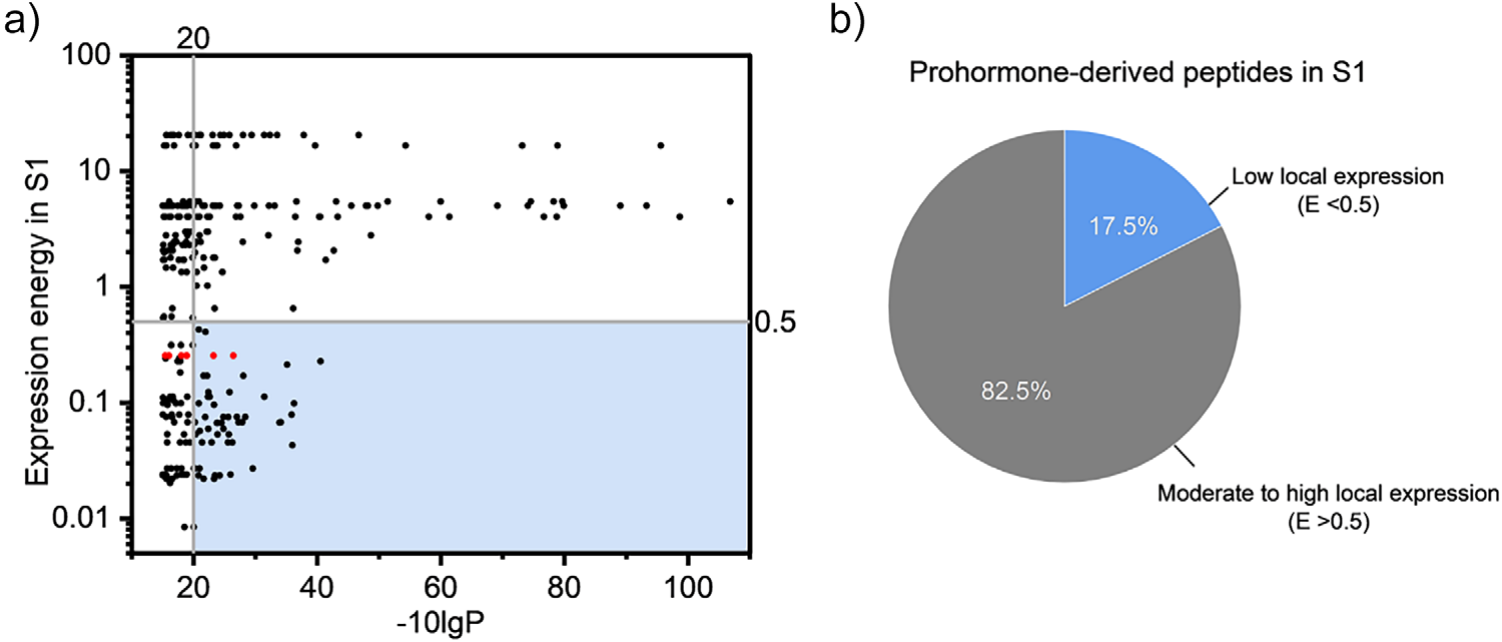
Classification of detected prohormone‐derived peptides based on local gene expression levels. a) Scatter plot of peptides’ ‐10LgP values versus their corresponding gene expression levels in the S1 region. The plot includes 305 prohormone‐derived peptides detected in S1 tissue samples, with expression energies shown on a logarithmic scale. The shaded blue region highlights peptides with low expression levels (E < 0.5) and high‐confidence matches (*p*  < 0.01). Red dots indicate POMC‐derived peptides. b) A pie chart showing the proportion of detected prohormone‐derived peptides with low local expression.

To better understand the origins of these prohormone‐derived peptides, we examined their local expression levels using ABADV (Figure 5). While the majority of detected peptides align with local gene expression levels, some exhibit minimal corresponding mRNA expression in S1. Among these low‐expression peptides, several are derived from POMC, with two identified with high confidence (Figure 5a, red dots). This finding aligns with prior observations in dialysate samples, further confirming their presence in this region despite minimal local *Pomc* mRNA expression. Given the extremely low local expression of *Pomc* mRNA (E = 0.25) and the lack of direct POMC neuronal innervation to the cortex, the most plausible explanation is that these peptides originate from extracellular bulk transfer rather than local synthesis. However, we cannot entirely rule out the possibility of low local expression or transport and release of POMC‐derived peptides in S1.

Beyond POMC‐derived peptides, several other peptides in the dataset also come from prohormones that are classically associated with subcortical regions. Although their local expressions are low (<0.5), these peptides are reliably identified in the S1 (Figure 5a), suggesting that they may originate from alternative sources. One explanation is the limited sensitivity of ISH at low expression levels—some peptides might be synthesized locally but remain undetected due to technical constraints.^[^ ^65^ ^]^ Alternatively, afferent projections could contribute to the presence of certain peptides. For example, galanin has a low expression level (0.17) in the S1 and is predominantly synthesized in several subcortical regions such as the hypothalamus and locus coeruleus (LC). A subset of noradrenergic neurons in the LC co‐express galanin and project broadly to the cerebral cortex,^[^ ^66^ ^]^ with galanin‐positive axons detected in the barrel field of S1.^[^ ^67^ ^]^ Although these findings imply galanin's involvement in sensory processing, its specific functions within the S1 cortex remain underexplored. Finally, extracellular bulk transfer through CSF may also play a role, as seen with beta‐endorphins,^[^ ^57^ ^]^ vasopressin,^[^ ^12^, ^68^ ^]^ and oxytocin.^[^ ^12^, ^69^ ^]^ Studies have shown that these peptides can be released into the ventricular system and exert effects at a different brain location. These observations suggest that multiple mechanisms may shape the extracellular peptide pool in cortical regions, providing a pathway for distant brain regions to modulate cortical sensory circuits.

Conversely, several identified prohormones are prominently expressed in the S1 cortex such as cholecystokinin (CCK), NPY, and somatostatin (SST). They are known to be highly expressed in specific subtypes of cortical interneurons,^[^ ^70^ ^]^ acting as neurotransmitters or neuromodulators to influence synaptic transmission.^[^ ^71^, ^72^, ^73^, ^74^ ^]^ Their functions in S1 are more characterized, in contrast to peptides transported from distant regions, which may have broader, less specific effects on cortical function.

Figure 5b provides a broader statistical perspective on the distribution of detected prohormone‐derived peptides, revealing that approximately 17.5% of identified peptides fall into the low‐expression category. This proportion suggests that a substantial subset of extracellular peptides in S1 may originate from non‐local sources, emphasizing the importance of considering bulk transfer when interpreting neuropeptide dynamics. Collectively, this complex and diverse peptidergic milieu underscores the multifaceted nature of neuropeptide signaling in S1, where locally produced and long‐range‐derived peptides likely converge to modulate synaptic transmission. These findings prompt further investigation into the cellular origins and functional roles of these peptides to determine whether they act as dynamic functional neuromodulators or simply represent products of prohormone processing.

Next, three additional in vivo samplings were performed, and the dialysate samples were analyzed with parallel reaction monitoring (PRM)‐PASEF. The previous DDA run from S1 tissue sample was used to construct a comprehensive spectral library. Peptides from secreted proteins were manually filtered again based on MS2 quality, and 258 of high confidence targets were compiled into a Skyline library for the PRM‐PASEF measurements and peptide match. The identified peptides are summarized in Table S1. A total of 46 peptides from 24 proteins were identified. Across the three biological replicates, peptides from a variety of neuropeptide precursors were identified, including calcitonin (CALC), NPY, proenkephalin‐A (PENK), and others. Two sequences PGMATLSEE (from CALC) and AAANFFRVLLQQLQMPQ (from corticotropin‐releasing factor, CRF), were shared between sample 2 and 3. Similarly, two overlapping sequences, YPSKPDNPGEDAPA and YPSKPDNPGEDAPAEDMARYYS from NPY, were detected in sample 1 and 3. These proteolytic variants of NPY may reflect differential processing events that could modulate its neuromodulatory functions. Proteolytic cleavage can generate fragments with distinct receptor affinities or terminate their bioactivities, potentially fine‐tuning the neuromodulatory effects of NPY in the brain. However, other detected peptides were unique to individual samples and did not overlap with those identified in our first biological sample analyzed using DDA. The observed variability in detected peptides across samples may reflect biological fluctuations in extracellular neuropeptide levels rather than just technical inconsistencies due to probe placement or limited sample volume. Neuropeptides are known to be highly dynamic, with release and degradation rates affected by neuronal activity, behavioral states, and metabolic conditions. While prior studies using larger sample volumes have reported broader peptide coverage, the majority do not specifically address extracellular peptides. For example, Raman et al.^[^ ^18^ ^]^ recently reported an interesting and novel membrane‐free sampling platform where 136 proteins were identified from three biological replicates. As many of these detected proteins are intracellular proteins, it suggests some tissue damage associated with probe implantation and sampling. In contrast, our method primarily detected secretory peptides, showing high specificity for extracellular peptide profiling.

Among the 24 detected proteins, 10 of them have mRNA expression levels lower than 0.5 (Table S2), highlighting the enhanced sensitivity of the new targeted method for detecting low‐abundance peptides. The presence of these peptides in S1 extracellular space may also reflect transport from distant sites through several mechanisms. Specifically, a few of them, including osteocrin (OSTN), pancreatic polypeptide prohormone (PPY), and brain natriuretic peptide (NPPB), had no observed mRNA expression throughout the mouse brain, suggesting possible transfer through the BBB from peripheral tissue^[^ ^75^, ^76^, ^77^, ^78^ ^]^; in fact, both PYY and NPPB are known to be found in the brain through such approaches. These findings further support the idea that cortical extracellular peptide pools may be shaped not only by local synthesis and release but also by long‐range molecular transport and highlight the need for such direct measurements as RNA‐based approaches would miss these molecules.

Looking ahead, we will refine both detection and quantification strategies to further uncover previously unrecognized aspects of neuropeptide‐mediated communication in the brain. Our system enables detection of low‐abundance peptides in limited dialysate samples, in line with previous studies that demonstrate the feasibility of analyzing peptides at low concentrations.^[^ ^79^, ^80^, ^81^, ^82^ ^]^ We have two areas that have potential for additional development. The next goal is to demonstrate quantitative capabilities, which we aim to address using isotopic standards. The standards can be added as internal standards for targeted peptides and added to the dialysate after sample collection. The standards serve as references for quantifying endogenous peptides using MS, improving the utility of our system for studying peptide dynamics in vivo. Lastly, our next iteration of the system will interface the output of our probe to a droplet generator^[^ ^34^, ^83^ ^]^ so that dynamic measurements can be made.

## Conclusion

This study unveils the intricate extracellular neuropeptide landscape of the mouse S1 using a novel membrane‐free ND probe coupled with LC‐MS. By eliminating the membrane, our design ensures unimpeded diffusion, facilitating the capture of a broader range of neurochemicals, including peptides and potentially proteins. The balanced in‐plane flow achieved with microfabrication techniques minimizes tissue damage and enhances spatial resolution.

Initial in vivo sampling identified 22 peptides from precursors such as secretogranin‐1, ProSAAS, and POMC, while subsequent targeted analyses across replicates revealed 46 peptides. Notably, the detection of POMC‐derived peptides, regardless of their relatively low expression in the cortex, suggests the platform's sensitivity in capturing molecules potentially originating from distant sources through bulk transfer mechanisms via CSF. Proteolytic variants of NPY suggest extracellular processing as a regulatory mechanism for diversifying neuromodulatory signals, potentially altering receptor specificity or bioactivity. Tissue peptidomics validated the existence of POMC‐derived peptides in S1 and further uncovered 304 prohormone‐derived peptides, with 17.5% originating from low‐expression precursors, implicating bulk transport or afferent projections in supplying cortical peptides. These findings position S1 as a convergence zone where locally generated peptides and distally transported signals may co‐regulate synaptic function, offering new insights into how neuropeptides modulate sensory integration beyond canonical roles. The probe's high specificity and minimal tissue disruption enabled precise detection of this diverse peptidome, offering insights into potential neuromodulatory roles in sensory integration. Future enhancements, including isotopic standards for quantification and droplet‐based dynamic sampling, are expected to deepen our understanding of neuropeptide functions in health and disease. This work establishes a robust framework for exploring the cortical extracellular neuropeptidome and its contributions to neural communication.

## Supporting Information

It contains experimental details, supplemental figure, and two tables. The authors have cited additional references within the Supporting Information.^[^ ^64^, ^84^, ^85^ ^]^

## Conflict of Interests

The authors declare no conflict of interest.

## Supporting information



Supporting information

Supporting information

Supporting information

## Data Availability

The MS peptidomics data have been deposited to the ProteomeXchange Consortium via the PRIDE^[^ ^86^ ^]^ partner repository with the dataset identifier PXD066497. All other data that support the findings of this study are available from the corresponding authors upon reasonable request.
