# Supplementary material for: Neuropeptides in the Extracellular Space of the Mouse Cortex Measured In Vivo by Nanodialysis Probe Coupled with LC‐MS
Source: Angew Chem Int Ed Engl. 2025 Aug 11;64(39):e202509490. doi: 10.1002/anie.202509490 (PMC12455431; doi:10.1002/anie.202509490)
Supplement: Supplementary file 1 — Supporting information [file ANIE-64-e202509490-s003.pdf]

Neuropeptides in the Extracellular Space of the Mouse Cortex Measured by In Vivo Nanodialysis Probe Coupled with LC-MS

Keyin Li[a] +, Weihua Shi[b] +, Yanqi Tan[d], Yu Ding[c], Alex G. Armstrong[a], Yurii Vlasov[a,b]*, Jonathan V. Sweedler[a,d]*

[a] K Li, A.G. Armstrong, J.V. Sweedler, Y. Vlasov
Neuroscience Program
University of Illinois Urbana-Champaign
Urbana, IL, 61801, USA

[b] W. Shi, Y. Vlasov
Department of Electrical and Computer Engineering
University of Illinois Urbana-Champaign
Urbana, IL, 61801, USA
E-mail: yvlasov@illinois.edu

[c] Y. Ding
Department of Physics, University of Illinois Urbana-Champaign
Urbana, IL, 61801, USA

[d] Y. Tan, J.V. Sweedler
Department of Chemistry and Beckman Institute for Advanced Science and Technology
University of Illinois Urbana-Champaign
Urbana, IL, 61801, USA
E-mail: jsweedle@illinois.edu

+These authors contribute equally to this work. *Corresponding authors

Supplemental Information

Experimental Details……………………………………………………………………………………………………. 2
Supplemental Table S1. List of peptides identified with PRM-PASEF………………………………………………………. 5
Supplemental Table S2. List of proteins identified with PRM-PASEF ……………………………………………………… 6
Supplemental Figure S1. In vitro relative recoveries of peptide standards…………………………………………………. 7

Experimental Details

Chemicals

Artificial cerebral spinal fluid (aCSF) was purchased from Tocris Bioscience, 1-Octanol was purchased from Thermo Scientific Chemicals, and all other reagents were purchased from Fisher Chemical.

Probe fabrication and operation

Starting from a custom-made SOI wafer (ULtrasil LLC) with 15 µm device layer, 1 µm oxide layer and 425 µm handle layer, a 300 nm silicon nitride (SiN_x) layer was first deposited on top of the silicon device layer as a hard mask via Oxford PECVD. A 0.5 µm AZ1505 photoresist (PR) layer was spin-coated for lithography. A series of 1X1 µm holes along the designed channel pathway were patterned by Heidelberg MLA150 direct laser writer, followed by development in AZ400T developer and Oxford Freon ICP RIE etching. Silicon etchant XeF_2 was then introduced to form isotropic channels through the 1 µm holes.

After channel formation, a thicker layer of 3 µm SiN_x deposition was performed in Oxford PECVD again to seal the channels. A 4 µm SPR220 PR layer is spin-coated and exposed in an EVG-620 mask aligner to define the device perimeters, followed by a 3 µm SiN_x etching in the Oxford Freon ICP RIE etching. Then the PR is stripped clean and spin-coated again with a fresh PR layer, with both the sampling area and the device perimeter patterned. A 20 µm Si etching in the STS Pegasus deep reactive ion etching (DRIE) and 1 µm oxide etching in the Oxford Freon ICP RIE is performed respectively, followed by another 3 µm SiN_x etching in the Oxford Freon ICP RIE. After these layers, the frontside devices are finished, with an open sampling area and defined device perimeters.

After the frontside fabrication, the wafer is flipped for backside patterning, while 425 µm silicon DRIE is performed in the designated probe and perimeter area, to release the device with a miniaturized in-vivo sampling probe.

After silicon microfabrication, each device is released and packaged individually. To interface with external pumps, the devices' outlet stubs are plumbed with Molex fused silica capillary (750 µm ID) using optical resin (Norland Optical Adhesive NOA 68T). For in vivo sampling, the aCSF perfusate was added to the inlet capillary with a Hamilton syringe and then sealed with a layer of 1-octanel. The outlet capillary was also added ~1 µL of 1-octanel to prevent evaporation during sample collection. Once the probe was prepared, they were connected to pressure-controlled pumps (Fluigent Flow-EZ, Push Pull) to supply pressure for flow rate control during the experiment.

***In vivo* sampling**

The animal subjects were adult Scnn1a-TG3-Cre x Ai32 mice (C57BL/6J origin), bred locally in the animal facility and housed under a 12 h light/dark cycle with free access to food and water. All procedures were approved by the Institutional Animal Care and Use Committee (IACUC) of the University of Illinois Urbana Champaign under protocol #23128, titled "Analysis of cortical circuits in behaving mice." All animal experiments were performed in accordance with the NIH Office of Laboratory Animal Welfare's Public Health Service Policy on Humane Care and Use of Laboratory Animals and Guide for the Care and Use of Laboratory Animals.

Isoflurane was used for all procedures requiring anesthesia, including surgeries and *in vivo* sampling experiments. Anesthesia was induced with 3% isoflurane and maintained at 1–2% throughout the procedures. Animals were kept on a heat pad to maintain body temperature, and ophthalmic ointment was applied to protect the eyes from desiccation.

For *in vivo* sampling in the mouse brain, the experiment began with headbar surgery. A titanium headbar was affixed to the skull to expose the left primary somatosensory area (S1). Sampling experiments were performed at least four days after the headbar surgery. On the day of the experiment, a small craniotomy was made over the targeted S1 region using a dental drill to enable the insertion of the sampling probe. The anesthetized mouse was head-fixed using the attached headbar, and the probe was mounted onto a motorized micromanipulator at a 38° angle. 10 µM of rhodamine B was added into the perfusate for visualization of the sampling site. The probe's push and pull channels were connected to pressure pumps via Teflon tubing for precise flow control. During insertion, both channels were backflushed with +100 mbar pressure to prevent tissue clogging. Once the probe reached the target depth of 500 µm, the flow configuration was switched to a push-pull mode with a flow rate of 10 nL/min. A single dialysate fraction (~1 µL) was collected over a 1.5–2 h period from each animal. For untargeted analysis, dialysate from one animal was used to create the peptide library. For targeted analysis, dialysate was collected from three animals and analyzed (n=3).

After sampling, the animal was sacrificed, and the brain was harvested. The brain was fixed in 4% paraformaldehyde at 4°C for a minimum of 24 h. The tissue was then sectioned into 50 µm slices using a Leica vibratome and imaged with a fluorescence microscope.

Sample preparation

During the *in vivo* sampling, the dialysate samples were collected in the silica capillaries and sealed with a layer of octanol to prevent evaporation. Once the sampling was completed, a Hamilton glass syringe (Hamilton 701N) was used to extract the sample. Due to the small sample volume (~1 µL), 5 µL of 20% acetonitrile was added to minimize sample loss during extraction. The entire aqueous phase was then transferred into a microcentrifuge tube for further processing. Residual octanol was removed by drying the sample in a vacuum concentrator (Genevac, Ipswich, UK), and the samples were subsequently cleaned using C18 ZipTip pipette tips (Millipore). The eluate was dried again and stored at -80 °C until LC-MS analysis.

For brain tissue samples, three mice were sacrificed by decapitation after anesthetizing with 5% isoflurane. The brains were extracted and the S1 parts were dissected. Tissues were immediately put on dry ice and then transferred to -80 °C until further processing. The brain samples were subjected to a three-stage peptide extraction adapted from Bora et al[84] and Anapindi et al[85]. First, each sample was weighed and homogenized in LC-MS grade water at a 10:1 (v/w) ratio, followed by incubation on ice for 40 minutes. After centrifugation at 16,000g for 20 minutes at 4°C, the supernatant was collected. The remaining pellet was resuspended in LC-MS grade 80/10/10 acetone/water/formic acid, incubated on ice for 40 minutes, and centrifuged under the same conditions. The second supernatant was collected. The pellet was then resuspended in LC-MS grade 0.25% formic acid in water, incubated on ice for 40 minutes, and centrifuged again at 16,000g for 20 minutes at 4°C. The final supernatants were collected, and all supernatants were combined and dried using a SpeedVac concentrator.

NanoLC-timsTOF data acquisition

The peptide extract from S1 tissues and dialysate samples were analyzed using a nanoElute LC system (Bruker Daltonics) coupled to a timsTOF Pro MS (Bruker Daltonics). The solvent A was 0.1% formic acid in LC-MS grade water and solvent B is LC-MS grade acetonitrile containing 0.1% formic acid. The peptides were separated on a nanoElute FIFTEEN column (Bruker Daltonics,75 µm x 150 mm, C18, 1.9 µm particles, 120 Å pore size). The separation was carried out at 40 °C with a flow rate of 300 nL/min using the following gradient: 2-10% B from 0-3 min, 10-40% B from 3-45 min, 40-90%B from 45-50min, followed by wash and equilibration steps. The LC was coupled to a timsTOF Pro MS (Bruker Daltonics) with a CaptiveSpray ion source. The MS was first operated in PASEF mode with a dynamic exclusion, 10 PASEF MS/MS scans per 1.9 s cycle. The m/z range was set between 100 and 1700, and the ion mobility range was set between 0.60 and 1.60 V·s/ cm^2 with a ramp time of 166 ms. The collision energy was ramped between 20.00 to 59.00 eV as a function of ion mobility.

Database search

The PEAKS Studio 10.6 (Bioinformatics Solutions Inc., Waterloo, Canada) was used for peptide identification through the database search function. The Uniport database for *mus musculus* was specified for all *in vivo* dialysate samples. The mass tolerance of the precursor ions was set to 20 ppm and product ions was 0.05 Da. No enzyme was selected for the database search. For the DDA analysis of the dialysate sample, the following variable modifications were included: oxidation (M), N-terminal acetylation, amidation (C), phosphorylation (STY), sulfation (STY), and pyro-glutamate formation from E and Q. For the DDA analysis of tissue samples, oxidation (M) and deamidation (NQ) were selected as variable modifications.

The mouse S1 tissue sample was searched against both the entire protein database and a list of prohormones.[64] A cutoff of 1% false discovery rate (FDR) was used for the search against the whole protein database. The FDR filter was turned off for prohormones to increase the peptide discovery rate and the -10LgP threshold was set to 15. The identified peptides were manually checked based on MS2 quality.

Targeted LC-MS/MS analysis

The method was built first by running the mouse cortex samples via DDA-MS. The acquired raw data from each LC-MS analysis was imported into PEAKS Studio X for peptide identification. The Mzxml and peptide-pepxml files were exported to Skyline (Version 23.1) software to build up a target peptide library, which consisted of individual peptide details including the amino acid sequence, precursor m/z values, and retention times. The raw DDA-MS files were imported into the Skyline project, and the ion mobility results from the cortex samples were added. The PRM-PASEF method including the precursor m/z values and ion mobility parameters was then exported from Skyline to the timsControl software on the instrument computer. The dialysate samples were dissolved in 2.5 µL of 0.1% formic acid in water, and 2 µL was injected. The same LC

settings, ion mobility parameters, and collision energy ramp as described for DDA-MS were applied. Eventually, the PRM-PASEF data was analyzed in Skyline, based on matching MS2 spectra to the library. Peptides were considered detected if the isotope dot product (idotp) score exceeded 0.8 and at least three fragment ions matched the reference spectrum. Due to the limited sample material, only a single measurement was performed for each variable from each subject.

Supplemental Table S1. List of peptides identified with PRM-PASEF

Sample 1	Sequence	Accession	Protein name
	NTTGPLQASNQ	P97297	ADML_MOUSE
	QDYMQMKAREL	P70160	CALC_MOUSE
	VMKCVLEVISDSLS	P26339	CMGA_MOUSE
	YKAIQKDDGQSDSQAV	P26339	CMGA_MOUSE
	LQDLALQGA	P26339	CMGA_MOUSE
	LQMPQRSL	Q8CIT0	CRF_MOUSE
	AATLEQPASAP	P22389	EDN2_MOUSE
	EPQSLPDYQFEKTE	Q9QY05	INSL6_MOUSE
	YPSKPDNPGEDAPA	P57774	NPY_MOUSE
	KRSFSGFGSPLD	P61364	OSTN_MOUSE
	TWVALLLQPLQGTWG	P10601	PAHO_MOUSE
	LGTGDNRAKDSHQQESTNNDED	P22005	PENK_MOUSE
	APSDPRLRQ	P60041	SMS_MOUSE
	SANSNPAMAPRE	P60041	SMS_MOUSE
	QARYKAARNQ	Q99ML8	UCN2_MOUSE
	AFAGQNSNTVLSRLLA	Q9QZQ3	UTS2_MOUSE
	ELERMALLQTLRQ	Q9QZQ3	UTS2_MOUSE
	IVNTNVPRASVPEG	P34884	MIF_MOUSE
	SLHSIGKIGGAQNRNYSKL	P34884	MIF_MOUSE
	GLLSDRLHISPDRVYINYYD	P34884	MIF_MOUSE
	GKLTGPSNQ	Q03517	SCG2_MOUSE
	HNGQVAEDAVSRPKD	Q0VGU4	VGF_MOUSE
	KKNAPPEPVPPPRAAPAPTHV	Q0VGU4	VGF_MOUSE
	RPASPPSVPGGSQQGTPEEA	Q0VGU4	VGF_MOUSE
**Sample 2**	LLFLYLSPLGGHS	P40753	ANFB_MOUSE
	PGMATLSEE	P70160	CALC_MOUSE
	APSGRMSVLKNLQSLDPSHRISD	P09240	CCKN_MOUSE
	KAPSGRMSVLKNLQSLDPSHRISD	P09240	CCKN_MOUSE
	R.KAPSGRMSVLKN.L	P09240	CCKN_MOUSE
	K.NLQSLDPSHRISD.R	P09240	CCKN_MOUSE
	AAANFFRVLLQQLQMPQ	Q8CIT0	CRF_MOUSE
	PETLISDLLMKESTENAPRTRLEDPS	P57774	NPY_MOUSE
	RYYSALRHYINLI	P57774	NPY_MOUSE
	INPLICSLECQD	O35417	PDYN_MOUSE
	R.SPQLEDEAKE.L	P22005	PENK_MOUSE
	Y.SKEVPEIEK.R	P22005	PENK_MOUSE
	QEQTHTAPAPW	P97297	ADML_MOUSE
	PRDNHLAPGQQTTLRIEGNQGAR	P01027	CO3_MOUSE
	SDRLHISPDRVYI	P34884	MIF_MOUSE
	GPEESREEVRFPD	P16014	SCG1_MOUSE
	TMEDHAGDYTLLQDQEGDMDHGLK	P10637	TAU_MOUSE
**Sample 3**	PGMATLSEE	P70160	CALC_MOUSE
	EKHLLLAALMQD	Q99MP3	CALCB_MOUSE
	AAANFFRVLLQQLQMPQ	Q8CIT0	CRF_MOUSE
	YPSKPDNPGEDAPAEDMARYYS	P57774	NPY_MOUSE
	DQDGNPL	O70176	PACA_MOUSE

Supplemental Table S2. List of proteins identified with PRM-PASEF

Protein name	Gene name	Expression in S1
CALCB_MOUSE	Calcb	0.00844
UTS2_MOUSE	Uts2	0.04548
INSL6_MOUSE	Insl6	0.059622
OSTN_MOUSE	Ostn	0.075316
PAHO_MOUSE	Ppy	0.09635
EDN2_MOUSE	Edn2	0.099262
ANFB_MOUSE	Nppb	0.106072
CALC_MOUSE	Calca	0.112872
CO3_MOUSE	C3	0.197818
ADML_MOUSE	Adm	0.241808
MIF_MOUSE	Mif	1.95274
CRF_MOUSE	Crh	2.05858
PDYN_MOUSE	Pdyn	2.79079
PACA_MOUSE	Adcyap1	2.98887
SCG2_MOUSE	Scg2	3.59481
SMS_MOUSE	Sst	4.03568
PENK_MOUSE	Penk	5.02753
NPY_MOUSE	Npy	5.47182
CCKN_MOUSE	Cck	16.6229
CMGA_MOUSE	Chga	20.5434
TAU_MOUSE	Mapt	21.9723
VGF_MOUSE	Vgf	25.9979
SCG1_MOUSE	Chgb	30.2193
UCN2_MOUSE	Ucn2	NA

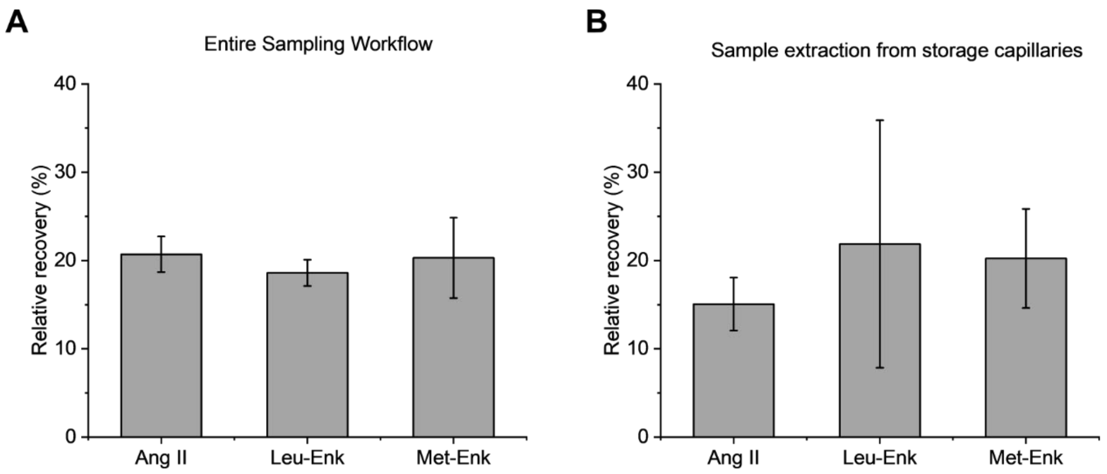

Supplemental Figure S1. *In vitro* relative recoveries of peptide standards angiotensin II (Ang II), leu-enkephalin (Leu-Enk), and met-enkephalin (Met-Enk). (A) Relative recoveries of peptide standards across the entire sampling workflow, including 1-hour microdialysis, sample extraction from storage capillaries, and transfer to MS analysis. (B) Relative recoveries of peptide standards from downstream extraction process to MS analysis.
