# Supplementary material for: Neuropeptides in the Extracellular Space of the Mouse Cortex Measured In Vivo by Nanodialysis Probe Coupled with LC‐MS
Source: Angew Chem Int Ed Engl. 2025 Aug 11;64(39):e202509490. doi: 10.1002/anie.202509490 (PMC12455431; doi:10.1002/anie.202509490)
Supplement: Supplementary file 2 — Supporting information [file ANIE-64-e202509490-s001.pptx]

## Slide 1
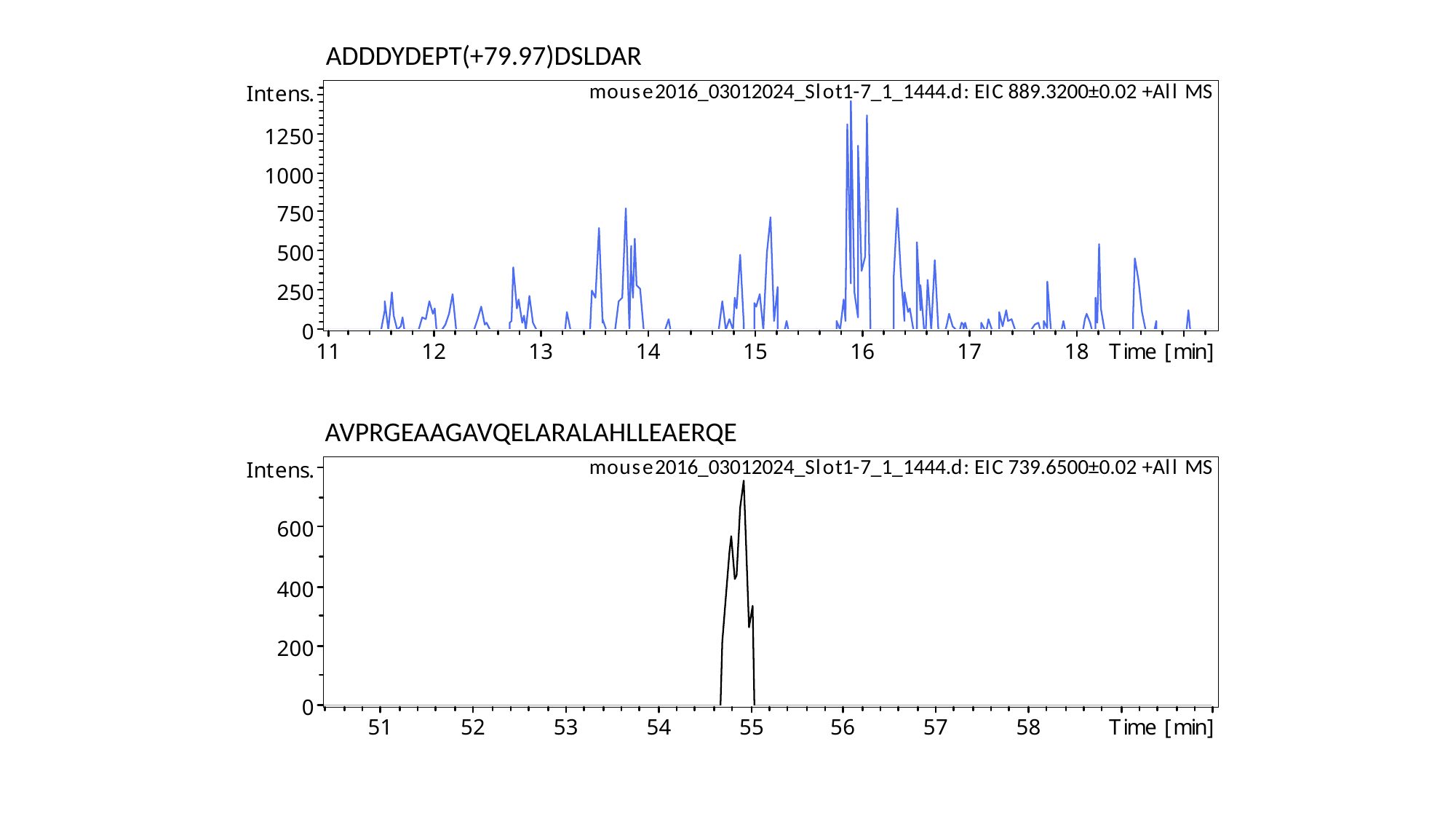

ADDDYDEPT(+79.97)DSLDAR
AVPRGEAAGAVQELARALAHLLEAERQE

## Slide 2
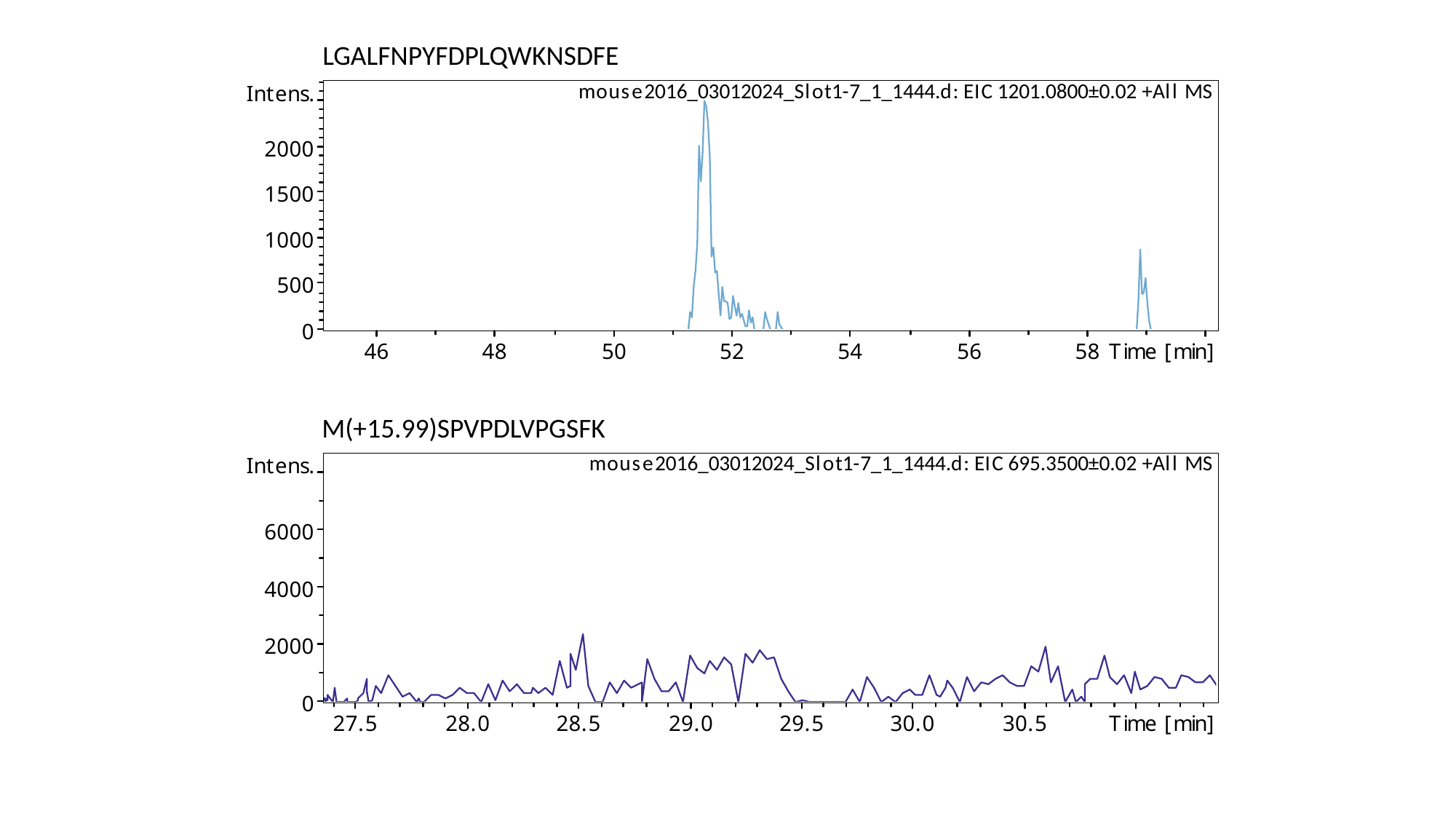

LGALFNPYFDPLQWKNSDFE
M(+15.99)SPVPDLVPGSFK

## Slide 3
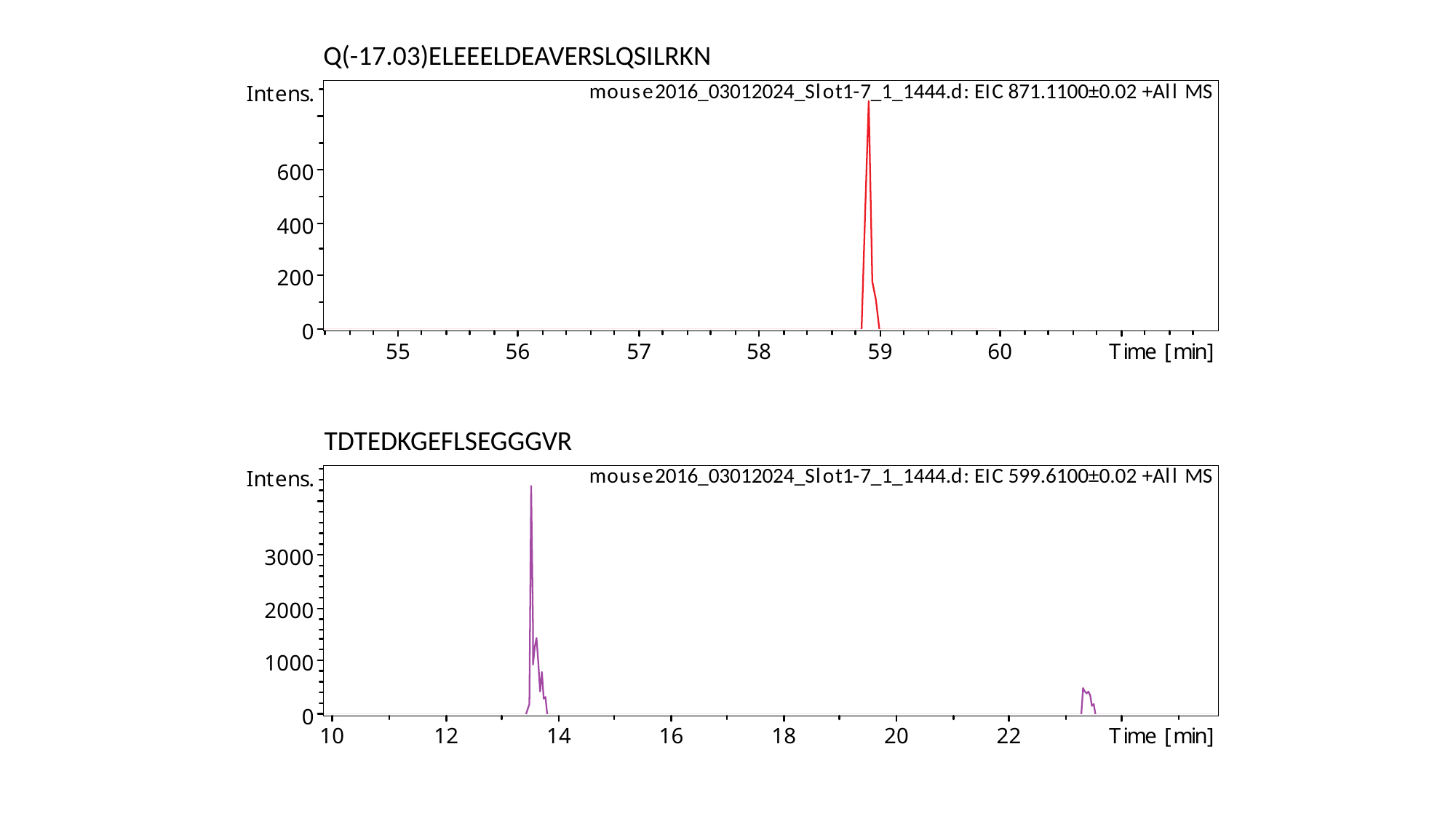

Q(-17.03)ELEEELDEAVERSLQSILRKN
TDTEDKGEFLSEGGGVR

## Slide 4
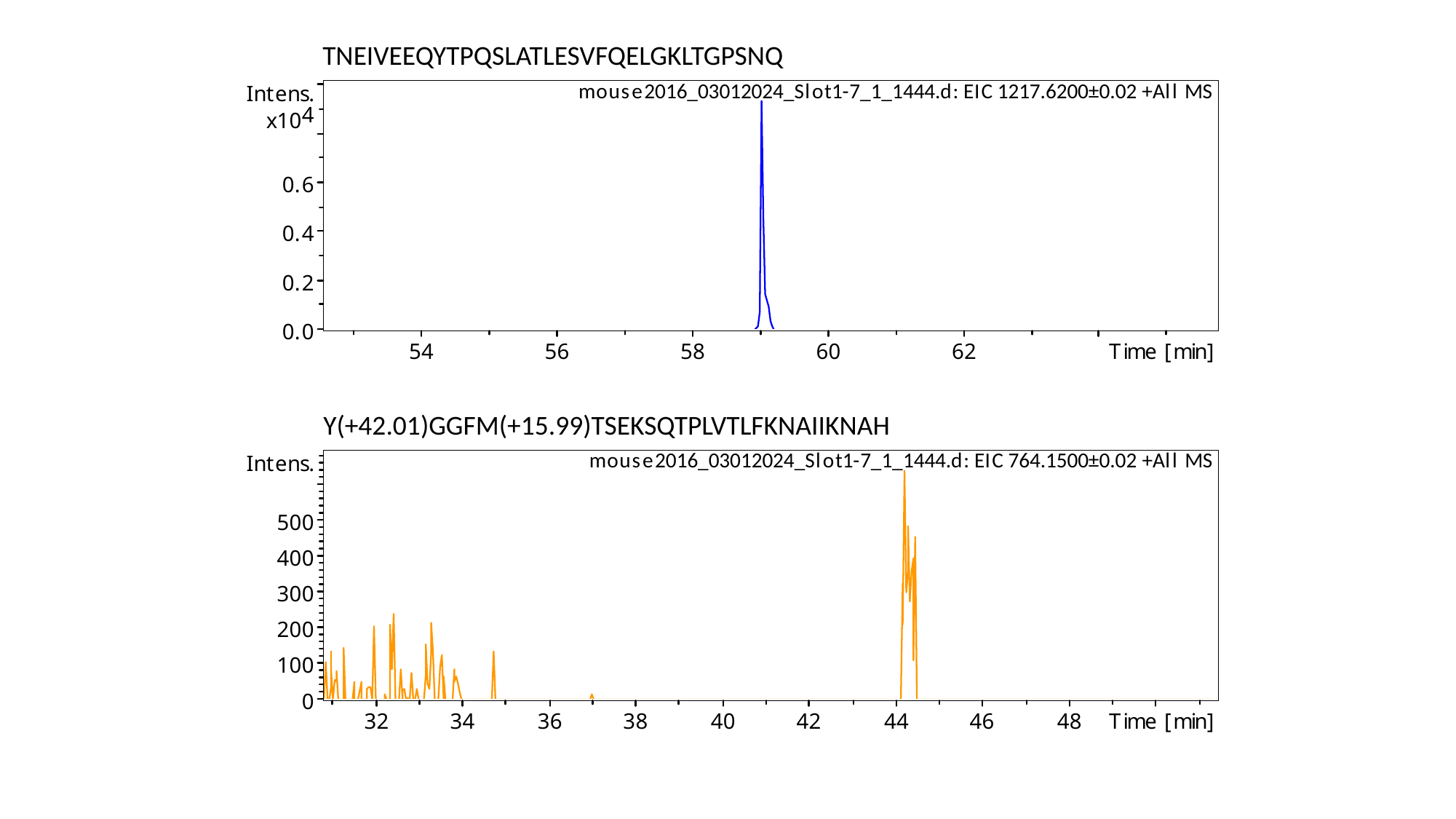

TNEIVEEQYTPQSLATLESVFQELGKLTGPSNQ
Y(+42.01)GGFM(+15.99)TSEKSQTPLVTLFKNAIIKNAH

## Slide 5
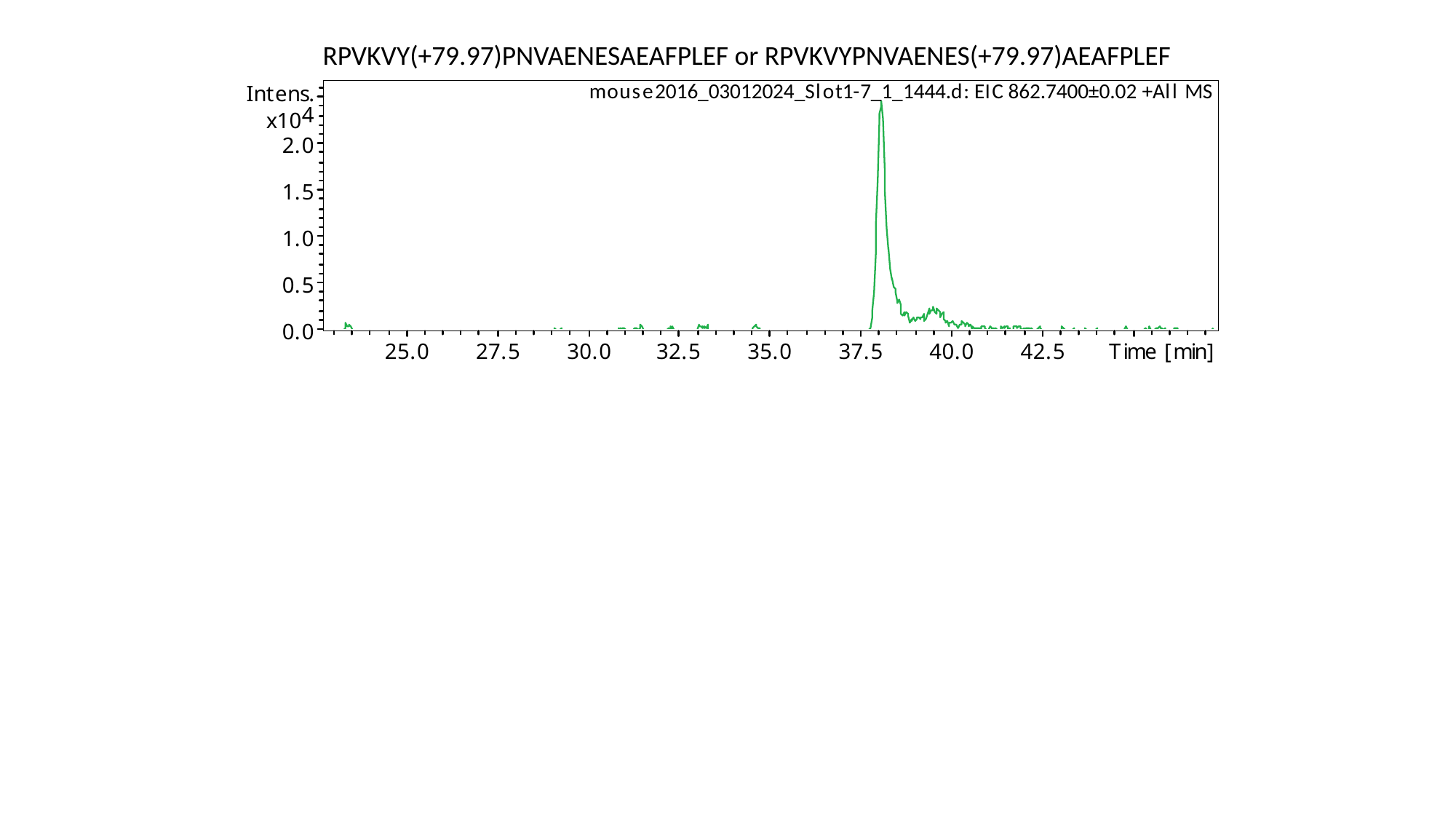

RPVKVY(+79.97)PNVAENESAEAFPLEF or RPVKVYPNVAENES(+79.97)AEAFPLEF
